# An in vitro study on *Staphylococcus schweitzeri* virulence

**DOI:** 10.1038/s41598-021-80961-x

**Published:** 2021-01-13

**Authors:** Almut Grossmann, Neele J. Froböse, Alexander Mellmann, Abraham S. Alabi, Frieder Schaumburg, Silke Niemann

**Affiliations:** 1 Institute of Medical Microbiology, University Hospital Münster, Münster, Germany; 2 Institute for Hygiene, University Hospital Münster, Münster, Germany; 3 Centre de Recherches Médicales de Lambaréné, Lambaréné, Gabon; 4 Section of Medical and Geographical Infectiology, University Hospital Münster, Münster, Germany

**Keywords:** Bacterial pathogenesis, Clinical microbiology

## Abstract

*Staphylococcus schweitzeri* belongs to the *Staphylococcus aureus*-related complex and is mainly found in African wildlife; no infections in humans are reported yet. Hence, its medical importance is controversial. The aim of this work was to assess the virulence of *S. schweitzeri *in vitro. The capacity of African *S. schweitzeri* (n = 58) for invasion, intra- and extracellular cytotoxicity, phagolysosomal escape, coagulase activity, biofilm formation and host cell activation was compared with *S. aureus* representing the most common clonal complexes in Africa (CC15, CC121, CC152). Whole genome sequencing revealed that the *S. schweitzeri* isolates belonged to five geographical clusters. Isolates from humans were found in two different clades. *S. schweitzeri* and *S. aureus* showed a similar host cell invasion (0.9 vs. 1.2 CFU/Vero cell), host cell activation (i.e. expression of pro-inflammatory cytokines, 4.1 vs. 1.7 normalized fold change in gene expression of *CCL5*; 7.3 vs. 9.9 normalized fold change in gene expression of *IL8*, A549 cells) and intracellular cytotoxicity (31.5% vs. 25% cell death, A549 cells). The extracellular cytotoxicity (52.9% vs. 28.8% cell death, A549 cells) was higher for *S. schweitzeri* than for *S. aureus*. Nearly all tested *S. schweitzeri* (n = 18/20) were able to escape from phagolysosomes. In conclusion, some *S. schweitzeri* isolates display virulence phenotypes comparable to African *S. aureus. S. schweitzeri* might become an emerging zoonotic pathogen within the genus *Staphylococcus*.

## Introduction

*Staphylococcus aureus*, *Staphylococcus argenteus* and *Staphylococcus schweitzeri* form the *S. aureus*-related complex^1^. The virulence and clinical outcome of *S. aureus* and *S. argenteus* is comparable but the capacity of *S. schweitzeri* to cause disease in humans remains controversial since no clinical infection has been reported, yet^2^. *S. schweitzeri* differs from *S. aureus* in the peptidoglycan type; the Genome-wide average nucleotide identity is only 88.6%^1^. In routine diagnostics, *S. schweitzeri* can be distinguished from *S. aureus* based on a *nuc*-PCR, NRPS-gene or by MALDI-TOF^2^.

*Staphylococcus schweitzeri* is mainly found as a colonizing strain of the nasopharynx in Afrotropical wildlife, particularly in bats and primates, but can also be detected on fomites^3,4^. So far, only three cases of a nasopharyngeal *S. schweitzeri* colonization in humans were reported from rural Gabon and might have been acquired while handling bushmeat^5,6^. *S. schweitzeri* can carry almost the same virulence factors as described for the other members of the *S. aureus*-related complex such as enterotoxins, toxic shock syndrome toxin, exfoliative toxins, leukocidins, hemolysins, adhesins, autolysins, immune evasion factors, or polysaccharide capsules^2^. A first functional study revealed that the *S. schweitzeri* type strain FSA084T produces α-hemolysin and is cytotoxic to human cell lines (HeLa, HT29)^7^. These preliminary results lead us to the hypothesis that the virulence of *S. schweitzeri* and *S. aureus* is equivalent. However, other functional aspects (e.g. invasion) of *S. schweitzeri* have not been studied, yet. Since virulence differs between isolates of various genetic backgrounds in in vitro studies, a representative strain collection of *S. schweitzeri* is needed. In addition, no clinical samples from humans in sub-Saharan Africa were so far screened systematically for *S. schweitzeri* to detect spill-over events from wildlife to humans.

Therefore, the aims of this study were (1) to test the virulence of *S. schweitzeri* in a wide range of in vitro assays on the largest *S. schweitzeri* collection published so far and (2) to screen systematically clinical samples from humans living in an area where *S. schweitzeri* is reported frequently in wildlife.

## Results

### Phylogeny

**Figure 1:**
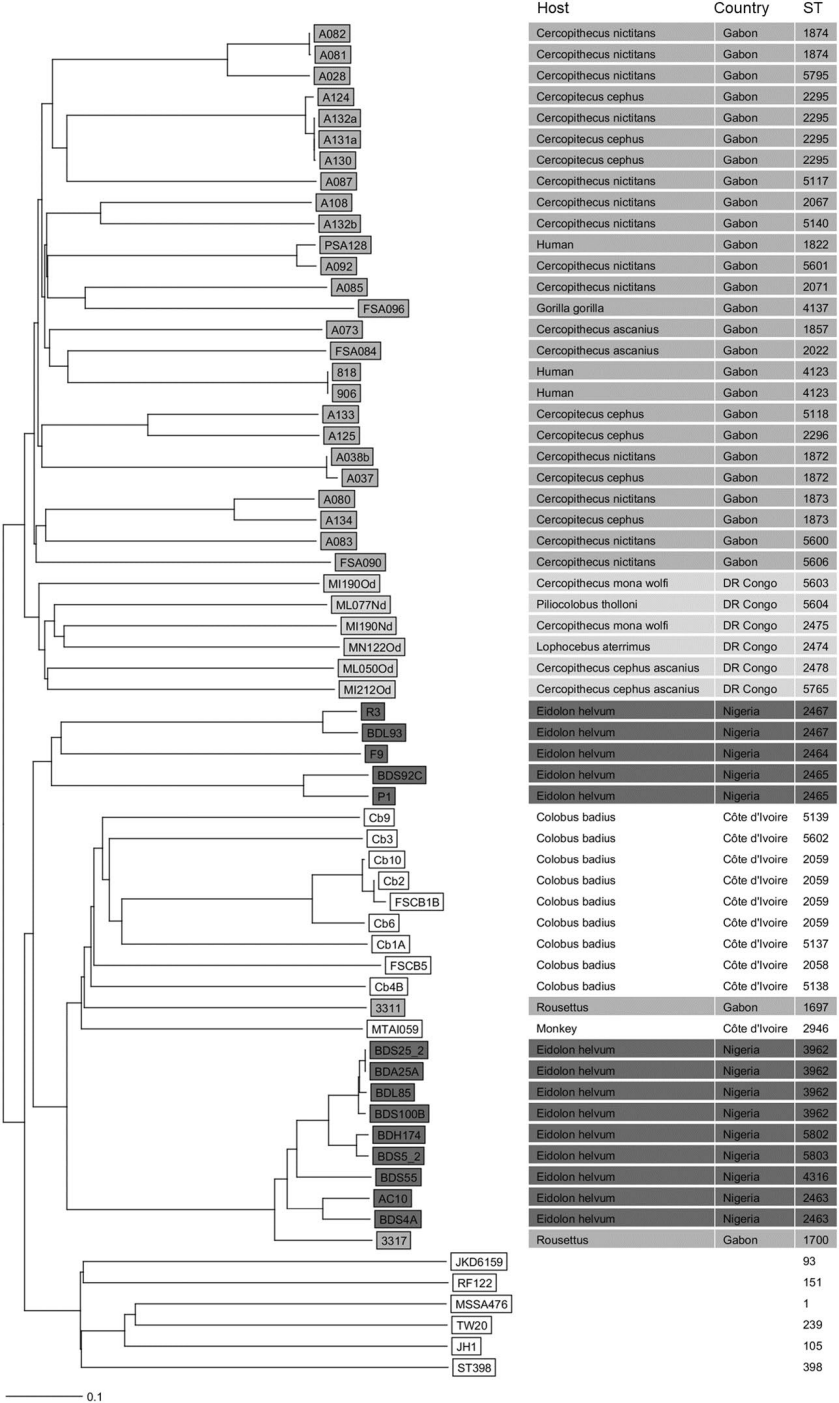
Neighbour-joining tree of *Staphylococcus schweitzeri*. The genomes of *S. schweitzeri* (n = 58) from African wildlife and humans in Côte d’Ivoire, Democratic Republic of the Congo, Nigeria and Gabon were sequenced and the phylogenetic tree was constructed based on the up to 1861 targets of the *S. aureus* cgMLST scheme^29^. MLST ST, host and country of origin are indicated for each isolate. The colour-code represents the country of origin. *S. aureus* strains JKD6159, RF122, ST398, JH1, MSSA476 and TW20 were selected for comparison^1^.

A total of 58 isolates from four African countries were included in the study and were confirmed to be *S. schweitzeri* as all clustered in a neighbor-joining tree based on the cgMLST scheme with the *S. schweitzeri* type strain (FSA084 = DSM 28300) separately from *S. aureus* standard strains (Fig. 1). *S. schweitzeri* isolates were grouped in five geographical clusters including two from Nigeria. The three *S. schweitzeri* from humans grouped in two separate clades from Gabon. The most closely related isolates of the human isolates were detected in monkeys (*C. nictitans*, *C. ascanius*).

### *S. schweitzeri* screening

To test if *S. schweitzeri* can cause clinical infection that may have gone unnoticed, we screened clinical isolates (n = 156) provisionally identified as *S. aureus* for characteristics of *S. schweitzeri* (i.e. negative *nuc*-PCR, 340 bp NRPS). These isolates are from the Lambaréné region in Gabon, where the only human carriers of *S. schweitzeri* were found so far. In total, the majority of isolates were from wound swabs (n = 43, 27.5%), followed by abscesses (n = 24, 15.4%) and skin and soft tissue infections (n = 22, 14.1%) and others (n = 67, 43.0%). All isolates were confirmed to be *S. aureus* (i.e. positive *nuc*-PCR, 160 bp NRPS). Thus, *S. schweitzeri* was not detected in human cases of infection.

### Coagulase assay

The majority of *S. schweitzeri* isolates was able to coagulate rabbit plasma (n = 57, 98%) followed by human plasma (n = 55, 95%) and chimpanzee plasma (n = 39, 67%). 39 isolates (67%) coagulated all three different plasma (Figure S1).

### Growth curves

**Figure 2:**
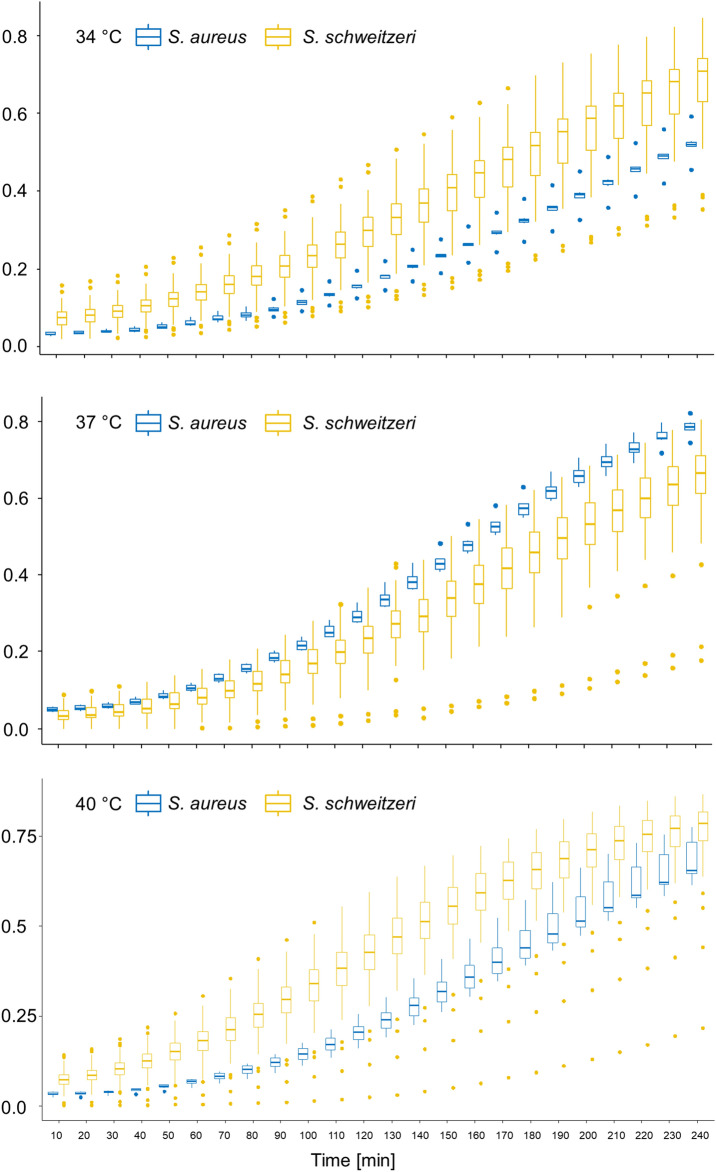
Growth curve of *Staphylococcus schweitzeri* and African *Staphylococcus aureus* at different temperatures. The growth of *S. schweitzeri* (n = 58, yellow boxplots) and a representative selection of African *S. aureus* (n = 6, blue boxplots) was photometrically measures after intervals of 10 min for 4 h. Boxplots (incl. outliers) of optical density (OD) are shown for each species and time points. *S. schweitzeri* grew faster at 34 °C and 40 °C than *S. aureus* comparator isolates.

*Staphylococcus schweitzeri* appeared to grow better than African *S. aureus* isolates at 34 °C and 40 °C, the corresponding body temperatures of bats and monkey, respectively (Fig. 2). A further statistical comparison of the growth curves using a two-way repeated measures ANOVA was not possible due to inhomogeneity of data on account of numerous extreme outliers in both groups (Fig. 2).

### Invasion of *S. schweitzeri* in epithelial cells of human and monkey origin

**Figure 3:**
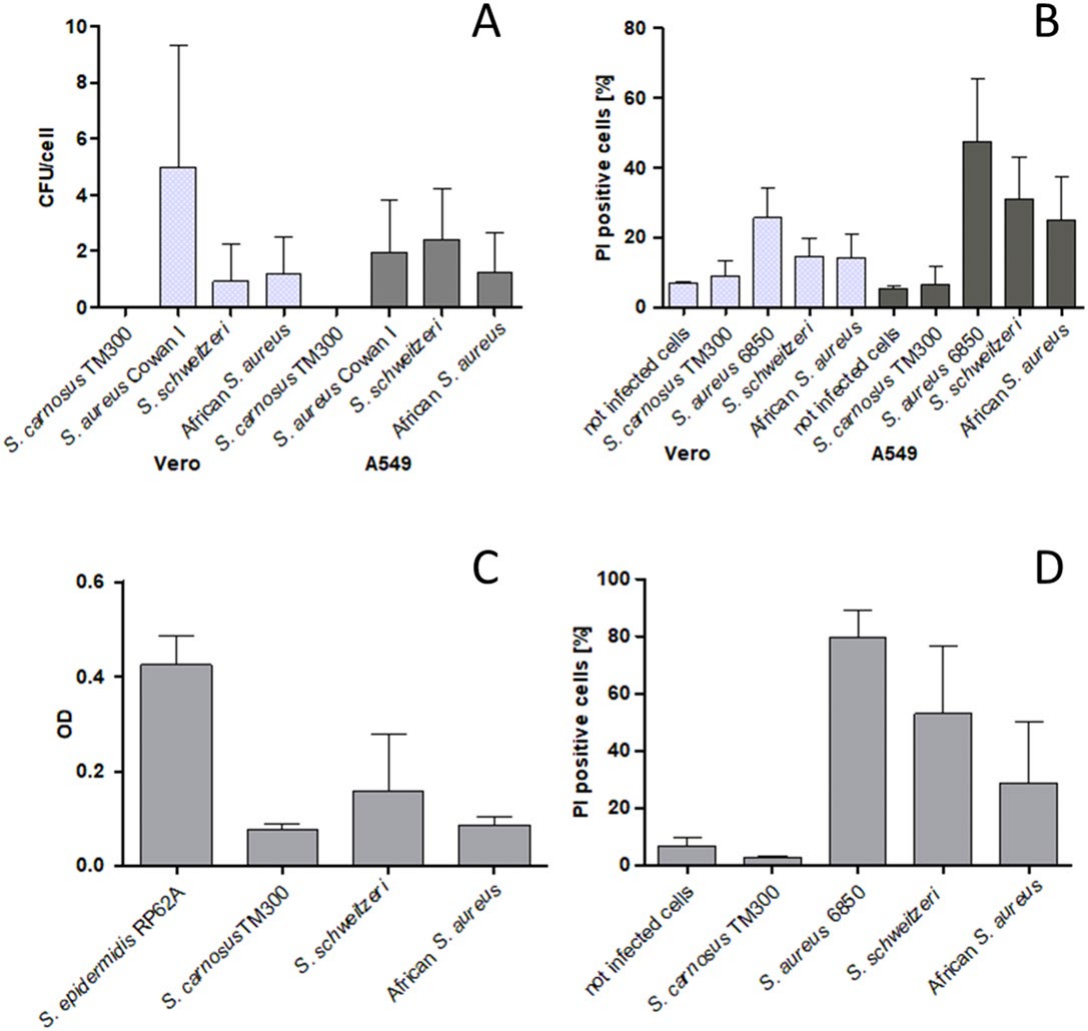
Comparison of the in vitro virulence of *Staphylococcus schweitzeri* and representative African *Staphylococcus aureus*. The results were merged for *S. schweitzeri* (n = 58) and African *S. aureus* (n = 6) and displayed as the mean (± SD) for each of the two groups. (**A**) Cellular invasion in Vero and A549 cells. The highly invasive strain *S. aureus* Cowan I served as positive and the low invasive *S. carnosus* strain TM300 as negative control. (**B**) Intracellular cytotoxicity after infection of Vero and A549 cells. The highly cytotoxic *S. aureus* strain 6850 served as positive and the *S. carnosus* strain TM300 as negative control. (**C**) Biofilm formation. The biofilm production was measured photometrically and bars indicated mean OD (± SD). (**D**) Extracellular cytotoxicity. A549 cells were exposed to secreted toxins in supernatants of overnight cultures. The mean cytotoxicity (proportion of dead cells, ± SD) of *S. schweitzeri* was significantly stronger than for African *S. aureus* (p = 0.02).

The ability to invade human epithelial lung cells (A549) and monkey kidney cells (Vero) was compared between *S. schweitzeri* and African *S. aureus* (Fig. 3A). The mean number of colony forming units (CFU) in Vero cells was 0.9 CFU/cells (*S. schweitzeri*) and 1.2 CFU/cells (African *S. aureus*). For A549 cells, *S. schweitzeri* was detected more often intracellularly than *S. aureus* (2.5 CFU/cells vs. 1.3 CFU/cells). However, if the ability to invade Vero cells and A549 cells is equivalent or statistically different in *S. schweitzeri* and *S. aureus* remained undetermined in the two one-sided test (TOST) procedure (Equivalence Test: p > 0.9, null hypothesis significance tests [NHST]: p > 0.1). While the African *S. aureus* seem to invade Vero and A549 cells in comparable numbers, *S. schweitzeri* was more often detected intracellularly in A549 cells compared to Vero cells (2.5 CFU/cells vs. 0.9 CFU/cells).

The ability to invade host cells varied markedly among the different *S. schweitzeri* isolates for A549 cells (range 0.2–7.9 CFU/cells) and Vero cells (range 0.1–6.9 CFU/cells, Figure S2). The majority of *S. schweitzeri* invaded human epithelial cells (A549) better than monkey kidney cells (Vero cells). The invasion of certain *S. schweitzeri* isolates was similar to African *S. aureus* strains.

### Intracellular cytotoxicity

The cytotoxicity (i.e. % of dead cells after infection) was noticeably similar for *S. schweitzeri* and African *S. aureus* in Vero cells (14.7% vs. 14.2%) and A549 cells (31.5% vs. 25%) but both the equivalence test and NHST were not statistically significant for Vero cells (p = 0.5 and p = 0.8, respectively) and A549 cells (p = 0.3 and p = 0.2, respectively). Therefore, it remains undetermined, if the intracellular cytotoxicity is equivalent or statistically different in *S. schweitzeri* and *S. aureus*.

Furthermore, the A549 cells were significantly more susceptible to intracellular cytotoxicity caused by *S. schweitzeri* than Vero cells (31.5% vs 14.7% p < 0.001, student’s t-test, Fig. 3B). Noteworthy, intracellular cytotoxicity differed markedly among the 58 *S. schweitzeri* isolates for A549 cells (range 8.1–58.5%) and Vero cells (range 8.3–39.0%, Figure S3).

### Phagolysosomal escape

Almost all randomly selected *S. schweitzeri* (18/20) were able to escape from phagolysosomes (mean: 1.82 bacteria/cell, Figure S4). The *S. aureus* strain 6850 and USA300 showed a phagosomal escape with a mean of 1.78 bacteria/cell and 1.8 bacteria/cell, respectively (Figure S4). However, we detected a large variance within the different *S. schweitzeri* isolates (range 0.1–3.5 escaped bacteria/cells, Figure S4).

### Expression of inflammation-related genes

The inflammatory response of A549 cells to an infection with 20 randomly selected *S. schweitzeri* and three African *S. aureus* isolates (one strain each of the three most common clonal MLST complexes in sub-Saharan Africa) was determined by the expression of *IL8* and *CCL5*. After 8 h, the expression levels (in normalized fold change of gene expression) were 4.1 ± 2.3 (*CCL5*) and 7.3 ± 4.0 (*IL8*) in cells infected with *S. schweitzeri* and 1.7 ± 0.5 (*CCL5*) and 9.9 ± 5.9 in cells infected with *S. aureus*. After the TOST procedure, it remained undetermined, if differences in the expression level of *CCL5* and *IL8* were different (p > 0.1) or equivalent (p > 0.4) after 8 h.

The expression of *IL8* was not different after 24 h in A549 cells infected with *S. schweitzeri* or *S. aureus*. In contrast, *CCL5* expression after 24 h was significantly higher (and not equivalent) in A549 cells infected with *S. schweitzeri* compared to A549 cells infected with African *S. aureus* (8.3 vs 1.8 normalized fold expression, p = 0.02, Figure S5). Similar to invasion, intracellular and extracellular cytotoxicity, the gene expression induced by *S. schweitzeri* infection varied among the 20 *S. schweitzeri* markedly (Figure S6).

### Biofilm

The thermostable nuclease (Nuc) plays a critical role in biofilm formation^8^. Since several polymorphisms of *nuc* are described for *S. schweitzeri*, we compared the biofilm formation between *S. schweitzeri* and *S. aureus*^9^. The ability to form biofilm was noticeably higher in *S. schweitzeri* than in African *S. aureus* (mean OD 0.16 vs. 0.09). Based on the TOST procedure it remains undetermined if the biofilm formation of *S. schweitzeri* vs. *S. aureus* is equivalent (p = 0.3) or statistically significantly different (p = 0.2). Both species were weak biofilm producers compared to the positive control *Staphylococcus epidermidis* RP62A (OD 0.42). *S. schweitzeri* showed a higher ability to form biofilm than the negative control *S. carnosus* TM300 (OD 0.08, p = 0.04, Fig. 3C). Genes associated with high formation of biofilm (i.e. *bap* and *icaA*), were not detectable in *S. schweitzeri* using whole genome sequencing. African *S. aureus* were tested positive for *icaA*, but also negative for *bap*.

### Extracellular cytotoxicity

As intracellular effects are only one aspect of virulence, we also investigated the cytotoxicity of secreted products of *S. schweitzeri* on A549 cells. The supernatant of overnight cultures of *S. schweitzeri* was significantly more cytotoxic compared to the African *S. aureus* strains (52.9% vs. 28.8%, Fig. 3D) in the TOST procedure (equivalence test: p = 0.7, NHST: p = 0.02). Supernatants of the negative control *S. carnosus* TM300 had no cytotoxic effect; supernatants of the positive control *S. aureus* 6850, on the other hand, had a very high cytotoxic effect. Similar to intracellular cytotoxicity, extracellular cytotoxicity differed strongly among the 58 *S. schweitzeri* isolates (range 6.4–89%, Figure S7).

## Discussion

We compared the in vitro virulence of *S. schweitzeri* with a selection of African *S. aureus* lineages and found that certain isolates of *S. schweitzeri* share similar virulence traits with African *S. aureus* strains.

The five phylogenetic clusters of *S. schweitzeri* rather correspond to the geographical origin than the host (Fig. 1). A definite delineation of geographical vs. host-related clusters is confounded by the fact that some host species are only found in one geographical area (e.g. *E. helvum* in Nigeria, *C. badius* in Côte d’Ivoire). However, geographical clusters of *S. schweitzeri* were also reported based on the *nuc* sequences^9^.

*Staphylococcus schweitzeri* was not detected in clinical samples in our study. Unlike colonization, there is no evidence that *S. schweitzeri* can cause infection in humans so far. However, the sole absence of *S. schweitzeri* in human infection should not be considered as a point neither for nor against its virulence. It might only be a snapshot of a temporal process. A cross-species transmission from wildlife to humans still remains possible since wildlife, particularly great apes or bats, are considered sentinels for the anticipation or early detection of zoonotic diseases in humans^10^.

While a coagulase activity for rabbit and human plasma was expected, the low numbers of isolates coagulating chimpanzee plasma (n = 39, 67%) was surprising. This could suggest a poorer adaptation of *S. schweitzeri* to great apes^11^. However, since great apes are the closest relatives to humans, one would expect a similar coagulase activity for plasma from humans and chimpanzees. A caveat for this comparison is that chimpanzee plasma was haemolytic (as observed by the naked eye through the change in plasma colour) indicative for a release of cellular molecules that interfere with coagulation (e.g. proteases, ADP) and only tested in monoplicates^12^.

*Staphylococcus schweitzeri* appeared to grow better at 34 °C and 40 °C than *S. aureus* (Fig. 2) suggesting that cell division of *S. schweitzeri* is more adapted to the mean body temperature of bats (34 °C) and monkeys (40 °C) than to humans^13^. This is in line with the observation, that *S. schweitzeri* is mostly found in bats and monkeys^2^.

Most of the *S. schweitzeri* strains invade both A549 and Vero cells (Fig. 3A, Figure S2). The invasion of *S. aureus* highly depends on the host cell type and the expression of virulence factors, which varies between strains^14^. One of the main factors for the uptake of *S. aureus* in host cells is the expression of fibronectin binding proteins A and B (FnBPA, FnBPB)^15^. The genes for one or both of these adhesins are also present in the genome of most of the *S. schweitzeri* isolates studied (Table S3). In contrast, all isolates lacked the gene for extracellular adherence protein Eap (also called Map), which, in addition to its adhesive and immunomodulatory properties, can also contribute to the invasion of *S. aureus* in host cells in the absence of FnBPs^16^. Interestingly, also in African *S. aureus* isolates only fragments and not the full *map* gene was detected^17^.

*Staphylococcus schweitzeri* can translocate from the phagolysosome into the cytoplasm after phagocytosis (Figure S3). The ability to escape from phagolysosome and to multiply in the cytoplasma of non-professional phagocytes eventually leading to cytolysis is now shown not only for *S. aureus* but also for *S. schweitzeri*^18,19^. The very low number of escaped bacteria/cell of some isolates (BDS92c, FSA096, P1) can possibly be attributed to the fact that these strains are only taken up to a small extent by the host cells (Figure S2).

The invasion of *S. aureus* into host cells triggers the release of pro-inflammatory cytokines^14^. This host defence can also exacerbate the course of the disease^20^. In the present study, we investigated the mRNA levels of *CCL5*- and *IL8* as examples for pro-inflammatory cytokines. CCL5 (RANTES) is chemotactic for T-cells, eosinophils, and basophils, and plays an active role in recruiting leukocytes into inflammatory sites. IL-8 (CXCL8) is a neutrophil chemoattractant that plays a central role in innate immunity to bacterial infection. Blood cells and many types of tissues produce IL-8. We showed that after infection with *S. schweitzeri* the host cells respond with a defense reaction, and the increase in cytokine expression is maintained for 24 h (Figure S6).

*Staphylococcus schweitzeri* produced more biofilm than the African *S. aureus* but the level of biofilm production was still markedly lower compared to the positive control *S. epidermidis* RP62A (Fig. 3C). Biofilm formation is regulated by numerous virulence factors such as the thermostable nuclease Nuc. Although the primary structure of Nuc from *S. schweitzeri* (*nuc*_*M*_) and *S. aureus* (*nuc1*) differ (identity 78.1–80.4%, similarity 92.4–94.1%), the nuclease activities are identical which is in line with a similar biofilm formation^9^. The weak biofilm formation by *S. schweitzeri* could be attributed to the lack of the biofilm producing genes *bap* or *icaA*. As *bap* was not detected in both *S. schweitzeri* and the African *S. aureus*, this could explain the weak biofilm formation. The African *S. aureus* was tested positive for *icaA*, but gene expression was not analysed.

Our study has some limitations. First, the African *S. aureus* controls showed low virulence compared to the positive controls in all assays. This is most likely due to the small sample size of this control group. Second, we saw differences in the cytotoxic effect of bacteria on human epithelial cells compared to monkey kidney cells. However, we cannot conclude that this is due to species adaptation as the cells are from different tissues (i.e. lung vs. kidney). Third, we applied in vitro assays, which give a hint but do not allow drawing definite conclusions about virulence in vivo. Further studies, also in animal models, are necessary to predict more accurately whether *S. schweitzeri* is pathogenic in humans or not. Fourth, although it is possible, our phylogenetic analysis does not allow for conclusions on transmission of *S. schweitzeri* between animals and humans. Further, particularly prospective observations are need to address transmission events. Fifth, due to the large sample size we did not adjust for the starting inoculum of the growth curves and used one colony of an overnight culture instead which might have affected the increase in growth. However, the small interquartile range at the first measuring point suggests that our approach most likely does not produce misleading results (Fig. 2).

In conclusion, the variance of *S. schweitzeri* in the applied virulence assays is large and certain isolates display virulence phenotypes comparable to African *S. aureus* isolates in the applied in vitro assays; however, clinical infections were not detected. *S. schweitzeri* might become an emerging zoonotic pathogen, if a cross-species transmission from African wildlife sustainably occurs.

## Materials and methods

### Ethical considerations

All clinical isolates from humans derived from the routine laboratory of the Albert Schweitzer Hospital in Lambaréné, Gabon. Informed consent was waived by the Institutional Ethics Committee of the “Centre de Recherches Médicales de Lambaréné [CERMEL]”)^21^. All methods were carried out in accordance with relevant guidelines and regulations of Gabon (https://cermel.org/ethicscommittee.php). All experimental protocols were approved by the Medical Faculty, Westfälische Wilhelms-Universität Münster, Germany (SC121720).

### Bacterial isolates

*Staphylococcus schweitzeri* isolates (n = 58) were collected in Gabon (n = 28), Nigeria (n = 14), Côte d’Ivoire (n = 10) and the Democratic Republic of the Congo (n = 6) between 2007 and 2017 (Table S1). They derived from monkeys (n = 38), bats (n = 16), humans (n = 3) and gorilla (n = 1)^4–6,22–24^.

The hypothesis of the study was that the virulence of *S. schweitzeri* and *S. aureus* is equivalent. Since *S. schweitzeri* is only found in Africa, we selected the most common African *S. aureus* lineages for reference and to control for a geographical bias. For that purpose, a total of six *S. aureus* reference strains from infection and colonization were selected from the three most common MLST clonal complexes (CC) in sub-Saharan Africa (CC15, CC121, CC152)^17,25^. These six reference strains are part of a strain collection from the Democratic Republic of the Congo but represent major sub-Saharan African clones^25^.

In preparation for the infection experiments bacteria from overnight culture in Tryptic Soy Broth (TSB) (shaking conditions, 37 °C) were adjusted to OD_578_ 0.1 in TSB. After 3 h of growth under the same conditions as before, the bacteria were adjusted to OD_578_ 1, and stored in aliquots at -20 °C until use. From a previously frozen aliquot the number of colony forming units (CFU) was determined after serial dilutions on blood agar of the bacterial suspensions and overnight incubation at 37 °C.

To test whether *S. schweitzeri* caused infections that may have gone unnoticed, clinical isolates (n = 156) provisionally identified as *S. aureus* (catalase- and coagulase Gram-positive cocci) at the Albert Schweitzer Hospital, Lambaréné, Gabon (2010–2016) were screened for *nuc*^26^ and the 340 bp-fragment of NRPS^1,27^. *S. schweitzeri* was defined by a negative *nuc*-PCR and the detection of the 340 bp-fragment of NRPS in catalase- and coagulase Gram-positive cocci^2^.

### Cell culture

We aimed to analyse the virulence using both human and monkey derived cell lines as *S. schweitzeri* is mainly found in monkeys and the *S. aureus* reference strains were from human origin. This selection was based on the assumption, that the two species were differently adapted to cell lines from different hosts. For that purpose, A549 cells (ACC 107, DSMZ GmbH, Braunschweig, Germany), a human alveolar epithelial cell line and Vero cells, a kidney epithelial cell line from monkeys were used (Vero cells were a gift from S. Ludwig, Institute of Molecular Virology, University of Münster). Cells were cultured in cell culture flasks (CELLSTAR, tissue culture-treated surface, Greiner Bio-One, Frickenhausen, Germany) in Dulbecco's modified Eagle medium (DMEM, Biochrom, Berlin, Germany), supplemented with 10% fetal calf serum (FCS, Biochrom, Berlin, Germany) at 37 °C and 5% carbon dioxide. For the infection experiments, A549 cells from passage 2 to passage 50 after defrosting from storage and Vero cells from passage 2 to 25 were used. Regular control experiments with well characterized *S. aureus* strains were used to check that cells from higher passages still showed the same behaviour as cells from the lower ones.

The cells were seeded at 40,000 cells/cm^2^ in 12 well plates (Corning,Costar, tissue culture-treated surface, Wiesbaden, Germany) 2–3 days before the experiment and were used at 90–100% confluence. To quantify the cell number, cells were seeded in an additional well in the tissue culture plate. On the day of the experiment, these cells were detached with trypsin–EDTA and the cell number was determined using an automated cell counter (TC20, Bio-Rad, Feldkirchen, Germany).

### Whole genome sequencing

Available *S. schweitzeri* sequences were retrieved from GenBank (accession nos. ERS140147, ERS140266, ERS140239, ERS140159, ERS140162, ERS140167)^1^. Sequences of African *S. aureus* reference strains were obtained from the European Nucleotide Archive (PRJEB15192)^25^. All other genomes were sequenced on a Illumina MiSeq platform (Illumina Inc., San Diego, USA) aiming for an at least 75-fold coverage^28^. Quality trimming and de novo assembly using the Velvet assembler was done with SeqSphere+ (version 5.9.0; Ridom GmbH, Münster, Germany)^28^.

Genomes were screened for MLST sequence types (ST) and 98 different virulence factors as described elsewhere^25^. A neighbor-joining tree was constructed based on up to the 1,861 genes of the *S. aureus* core genome (cg)MLST scheme^29^. New reads were deposited at the European Nucleotide Archive (accession no: PRJEB35847).

### Coagulase assay

Plasma from rabbits (bioMérieux, Marcy l’Etoile, France), humans (pooled from 10 different human samples, blood bank of the University Hospital Münster) and chimpanzees (pooled from seven chimpanzee samples, Allwetterzoo Münster, Münster, Germany, originally taken for veterinary purposes) were used to test the coagulase activity of *S. schweitzeri* towards different species (in triplicates). Due to the little volume of chimpanzee plasma, the test was performed only once. Bacterial cells from overnight cultures (1 × 10^8^) were suspended in 300 µl of the respective plasma and incubated for 4 h at 37 °C. A coherent clot when tilting the tubes was considered a positive result^11^. *S. aureus* ATCC 29213 and *S. epidermidis* ATCC 12228 were positive and negative controls, respectively.

### Growth curves

Growth curves of *S. schweitzeri* (n = 58) were photometrically measured (578 nm, Bio-Rad, Hercules, CA, USA) in triplicates every ten minutes for 4 h at 34 °C, 37 °C and 40 °C to assess an adaptation to different body temperatures of the different hosts (bats 34.8–37 °C, humans 37 °C, monkeys 40 °C)^13^. For this, one colony of overnight cultures was suspended in 200 µl TSB and incubated in 96-well plates (flat bottom). Wells were shaken briefly before each measurement.

### Invasion assay

A549 and Vero cells were infected with a multiplicity of infection of 50 (MOI50) in invasion medium (DMEM, 10% human serum albumin, 10 mmol/l HEPES, Merck, Berlin, Germany) and incubated for 3 h at 37 °C. The highly invasive strain *S. aureus* Cowan I served as positive and the low invasive *S. carnosus* strain TM300 as negative control^14,15^. After washing, extracellular bacteria were eliminated by lysostaphin (20 mg/ml, WAK-Chemie Medical, Steinbach, Germany) for 30 min. Cells were detached by EDTA-trypsin (Biochrom, Berlin, Germany). The number of cells was determined using an automated cell counter (BIO-RAD, TC20). Immediately afterwards, the cells were centrifuged and the pellet was taken up in ice-cold water for lysis of the cell. To determine the CFU, serial dilutions of the cell lysates were cultured overnight on Columbia blood agar at 37 °C. Invasion was expressed as the mean number of CFU per host cell.

### Intracellular cytotoxicity

The infection of Vero and A549 cells was performed as for invasion assays. The highly cytotoxic *S. aureus* strain 6850 served as positive and the *S. carnosus* strain TM300 as negative control^14^. Extracellular bacteria were removed by lysostaphin 3 h post infection. After overnight incubation (in DMEM, 10% FCS, Penicillin, Streptomycin, Merck, Berlin, Germany), cells were washed with PBS and adherent cells were detached with EDTA-trypsin. Supernatant, PBS from the washing step and detached cells from one well were collected in one tube, so that cells already detached by cell damage or cell death were included in the analysis. Dead host cells were stained with propidium iodide (PI, PD Pharmingen, Heidelberg, Germany) and analysed by flow cytometry. Cytotoxicity was expressed as the proportion of dead (i.e. % of PI-positive) cells.

### Phagolysosomal escape

Twenty *S. schweitzeri* isolates were randomly selected (https://www.randomizer.org/) to analyse the phagolysosomal escape^14^. Briefly, A549 cells were used which express the escape marker YFP-CWT in the cytoplasm. YFP-CWT is recruited to the peptidoglycan of the bacterial cell wall upon rupture of the phagolysosomal membrane barrier^30^. Subsequently, fluorescent rings around the bacteria become visible and allow the quantification of bacteria that have escaped into the cytoplasm. A549 YFP-CWT cells were infected in imaging dishes with cover glass bottom (MoBiTec, Göttingen, Germany) with *S. schweitzeri* for 1 h, followed by lysostaphin treatment and adding of fresh culture medium. After 2.5 h, cells were analysed by fluorescence microscopy (Axio Observer.Z1, Carl Zeiss, Jena, Germany, Zeiss filter set 38 HE, 100x/NA 1.3 plan-neofluar objective). Pictures were documented with an AxioCam MRm camera and processed using Zeiss AxioVision. Ten fields of view were analysed for each isolate (Figure S8) and the phagolysosomal escape was expressed as the mean number (± SD) of escaped bacteria per cell from three independent experiments. We defined that we do not consider an escaped bacteria/cell ratio < 0.5 as a phagolysosomal escape.

### Quantitative real-time-polymerase chain reaction (qRT-PCR)

qRT-PCR was used to analyse the gene-expression of inflammation-related *CCL5* and *IL8* to determine the activation of A549 host cells after infection with 20 randomly selected *S. schweitzeri*^14,31^ and 3 African *S. aureus* isolates from colonisation (one strain each of the three most common clonal MLST complexes in sub-Saharan Africa). Infection of A549 with *S. aureus* 6850 served as a control.

Infection was performed as described for intracellular cytotoxicity. At 8 and 24 h post infection, cells were detached with EDTA-trypsin, centrifuged, suspended in RNAprotect (QIAGEN, Hilden, Germany) and stored at − 20 °C. After RNA extraction (RNeasy Mini kit, QIAGEN), the concentration of total RNA was determined using a Nanophotometer (Implen, Munich, Germany) and adjusted to 1000 ng. RNA quality was assessed by determining the A260/A280 ratio. This ratio was between 1.7 and 2, with most samples showing a ratio around 1.9. cDNA was synthetized (Quantitect reverse transcription kit, QIAGEN) following the manufacturer’s recommendations and stored at − 80 °C.

Real-time amplification was done with the iQ SYBR Green Supermix (Bio-Rad) and specific primers (*GAPDH, B2M, CCL5*, *IL8*, Table S2) on an iCycler iQ real-time PCR-system (Bio-Rad), adding 50 ng cDNA to the reaction. The reaction mixtures were incubated for 15 min at 95 °C followed by 40 amplification cycles (15 s at 95 °C, 30 s at 55 °C, 30 s at 72 °C). iQ5 software was used to calculate PCR efficiencies, melting-curve analysis, and expression rates. *B2M* and *GAPDH* were used as endogenous controls to normalize expression levels^32^. The reference genes were chosen based on our previous study^14^. Data are presented as normalized fold change in expression compared to controls (non-infected cells) using the ∆∆Ct method. *S. aureus* strain 6850 was used as positive control.

### Biofilm formation

Overnight BHI-cultures were diluted (200-fold in BHI, including 0.25% glucose) in 96-well plates (flat bottom, Greiner bio-one, Frickenhausen, Germany) and incubated overnight at 37 °C. Cultures were washed twice (PBS) and stained with 1% crystal violet (Labochem international, Heidelberg, Germany) for 15 min. After washing (PBS, three times), the biofilm was dissolved in 100 µl ethanol-acetone (80:20) and the biofilm mass was measured photometrically (655 nm) in a microtiter plate reader (Bio-Rad). Controls were *S. epidermidis* strain RP62A (positive control) and *S. carnosus* TM300 (negative control).

### Extracellular cytotoxicity

A549 cells were seeded in 12-well plates two days before infection. Supernatants of bacterial overnight cultures (in TSB, rotational shaking at 37 °C) were sterile-filtered (0.22 µm; Millipore, Bedford, MA). A549 cells were incubated with 80% invasion medium and 20% filtered supernatant for 24 h. Cells were washed with PBS and adherent cells were detached with EDTA-trypsin. Supernatant, PBS from the washing step and detached cells from one well were collected in one tube, so that cells already detached by cell damage or cell death were included in the analysis. PI-stained cells were detected using flow cytometry. Cytotoxicity was expressed as the proportion of dead (i.e. % of PI-positive) cells.

### Statistics

All statistical analyses were done with “R” applying a significance level of 0.05^33^. Continuous variables (e.g. cytotoxicity, invasion, absorption in growth assay) were tested for normal distribution using a quantile–quantile plot (Q–Q plot) with a 45° reference line and the Shapiro–Wilk test. Outliers and extreme outliers were identified using the package “rstatix”. Only if the assumption of normality and homogeneity are met, the two-way repeated measures ANOVA was used to compare the growth curves of *S. schweitzeri* and *S. aureus*.

Means of normally distributed continuous variables (i.e. invasion, cytotoxicity, biofilm formation) from *S. schweitzeri* and *S. aureus* were tested for equivalence with the TOST procedure as implemented in the package “TOSTER”. This package includes both a null hypothesis significance test (NHST) and equivalence test. A comparison of the effects measured in the virulence assays between *S. schweitzeri* and *S. aureus* (Fig. 3) can remain undetermined if the effects are neither statistically different from zero nor statistically equivalent^34^. We set the equivalence bounds for large effects (Cohen’s d = 0.8)^35^.

Where indicated, selected comparisons of continuous variables were done using the two-tailed student’s t-test. For individual isolates (see supplement) medians and ranges are reported.

The datasets generated during and/or analysed during the current study as well as all bacterial strains are available from the corresponding author on reasonable request.

## Supplementary Information

Supplementary Information 1.

Supplementary Information 2.
