# Supplementary material for: An in vitro study on Staphylococcus schweitzeri virulence
Source: Sci Rep. 2021 Jan 13;11:1157. doi: 10.1038/s41598-021-80961-x (PMC7806826; doi:10.1038/s41598-021-80961-x)
Supplement: Supplementary file 1 — Supplementary Information 1. [file 41598_2021_80961_MOESM1_ESM.pdf]

Supplementary Material

An *in vitro* study on *Staphylococcus schweitzeri* virulence

Almut Grossmann[a], Neele J. Froböse[a], Alexander Mellmann[a,b], Abraham S. Alabi[c], Frieder Schaumburg[a,§,*], Silke Niemann[a,d,§]

[a]Institute of Medical Microbiology, University Hospital Münster, Münster, Germany

[b]Institute for Hygiene, University Hospital Münster, Münster, Germany

[c]Centre de Recherches Médicales de Lambaréné, Lambaréné, Gabon

[d]Section of Medical and Geographical Infectiology, University Hospital Münster, Münster, Germany

§contributed equally to this work

*address correspondence to Frieder Schaumburg, frieder.schaumburg@ukmuenster.de

Table S1: *Staphylococcus schweitzeri* strains of the study

ID	**Host**	**Country**	**Reference**
BDH174	*E. helvum*	Nigeria	Olatimehin, et al. [1]
BDL85	*E. helvum*	Nigeria	Olatimehin, et al. [1]
BDL93	*E. helvum*	Nigeria	Olatimehin, et al. [1]
BDS4A	*E. helvum*	Nigeria	Olatimehin, et al. [1]
BDS5_2	*E. helvum*	Nigeria	Olatimehin, et al. [1]
BDS25_2	*E. helvum*	Nigeria	Olatimehin, et al. [1]
BDA25A	*E. helvum*	Nigeria	Olatimehin, et al. [1]
BDS55	*E. helvum*	Nigeria	Olatimehin, et al. [1]
BDS92C	*E. helvum*	Nigeria	Olatimehin, et al. [1]
BDS100B	*E. helvum*	Nigeria	Olatimehin, et al. [1]
A124	*C. cephus*	Gabon	Schaumburg, et al. [2]
A125	*C. cephus*	Gabon	Schaumburg, et al. [2]
A083	*C. nictitans*	Gabon	Schaumburg, et al. [2]
FSA090	*C. nictitans*	Gabon	Tong, et al. [3]
A092	*C. nictitans*	Gabon	Schaumburg, et al. [2]
A028	*C. nictitans*	Gabon	Schaumburg, et al. [2]
MI190Od	*C. mona wolfi*	DRC	Schaumburg, et al. [2]
A037	*C. cephus*	Gabon	Schaumburg, et al. [2]
A038b	*C. nictitans*	Gabon	Schaumburg, et al. [2]
A073	*C. ascanius*	Gabon	Schaumburg, et al. [2]
A080	*C. nictitans*	Gabon	Schaumburg, et al. [2]
A081	*C. nictitans*	Gabon	Schaumburg, et al. [2]
A082	*C. nictitans*	Gabon	Schaumburg, et al. [2]
FSA084	*C. ascanius*	Gabon	Schaumburg, et al. [2]
MI190Nd	*C. mona wolfi*	DRC	Schaumburg, et al. [2]
A085	*C. nictitans*	Gabon	Schaumburg, et al. [2]
A087	*C. nictitans*	Gabon	Schaumburg, et al. [2]
FSA096	*G. gorilla*	Gabon	Schaumburg, et al. [2]
A108	*C. nictitans*	Gabon	Schaumburg, et al. [2]
FSCB1B	*C. badius*	IC	Tong, et al. [3]
Cb2	*C. badius*	IC	Tong, et al. [3]
Cb10	*C. badius*	IC	Tong, et al. [3]
Cb1A	*C. badius*	IC	Tong, et al. [3]
MI212Od	*C. ascanius*	DRC	Schaumburg, et al. [2]
Cb6	*C. badius*	IC	Tong, et al. [3]
Cb3	*C. badius*	IC	Tong, et al. [3]
Cb4B	*C. badius*	IC	Tong, et al. [3]
Cb9	*C. badius*	IC	Tong, et al. [3]
FSCB5	*C. badius*	IC	Tong, et al. [3]
MN122Od	*L. aterrimus*	DRC	Tong, et al. [3]
ML077Nd	*P. tholloni*	DRC	Tong, et al. [3]
ML050Od	*C. ascanius*	DRC	Tong, et al. [3]

A130	*C. cephus*	Gabon	Tong, et al. [3]
P1	*E. helvum*	Nigeria	Akobi, et al. [4]
F9	*E. helvum*	Nigeria	Akobi, et al. [4]
R3	*E. helvum*	Nigeria	Akobi, et al. [4]
AC10	*E. helvum*	Nigeria	Akobi, et al. [4]
MTAI59	Monkey	IC	Tong, et al. [3]
A131a	*C. cephus*	Gabon	Tong, et al. [3]
PSA128	Human	Gabon	Ateba Ngoa, et al. [5]
A133	*C. cephus*	Gabon	Own collection
3317	*Rousettus*	Gabon	Held, et al. [6]
3311	*Rousettus*	Gabon	Held, et al. [6]
A134	*C. cephus*	Gabon	Own collection
906	Human	Gabon	Okuda, et al. [7]
818	Human	Gabon	Okuda, et al. [7]
A132a	*C. nictitans*	Gabon	Schaumburg, et al. [2]
A132b	*C. nictitans*	Gabon	Schaumburg, et al. [2]

Note: DRC: Democratic Republic of the Congo, IC: Côte d’Ivoire

Table S2: Primers for qRT-PCRs

Gene	Forward primer	Reverse primer	Reference
CCL5	5'-CAGTGGCAAGTGCTCCAACC-3'	5'-CCATCCTAGCTCATCTCCAAAGAGT-3'	Strobel, et al. [8]
IL8	5'-CTTGTTCCACTGTGCCTTGGTT-3'	5'-GCTTCCACATGTCCTCACAACAT-3'	Voss, et al. [9]
B2M*	5'-TGAGTATGCCTGCCGTGTGA-3'	5'-AAATGCGGCATCTTCAAACCT-3'	Strobel, et al. [8]
GAPDH*	5'-GCAAATTTCCATGGCACCGT-3'	5'-GCCCCACTTGATTTTGGAGG-3'	Strobel, et al. [8]

*housekeeping genes

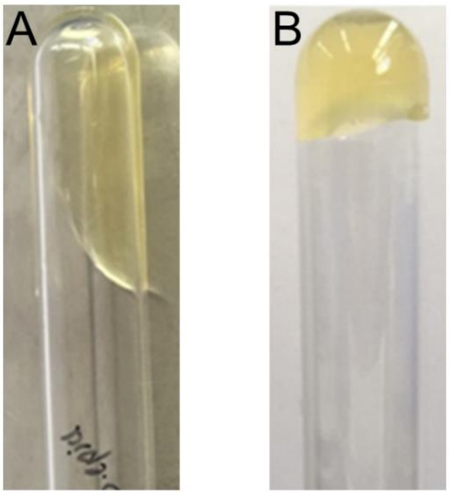

Figure S1: Coagulase assay: The presence of bacterial coagulase in *Staphylococcus schweitzeri* was tested by using bacterial cells from overnight cultures and suspend them in plasma from three different species (human, rabbit, chimpanzee). The bacteria were incubated for 4 hours at 37°C in the plasma. A liquid result was considered a negative result (A). A coherent clot when tilting the tubes was considered a positive result (B). *S. aureus* ATCC 29213 was used as positive control.

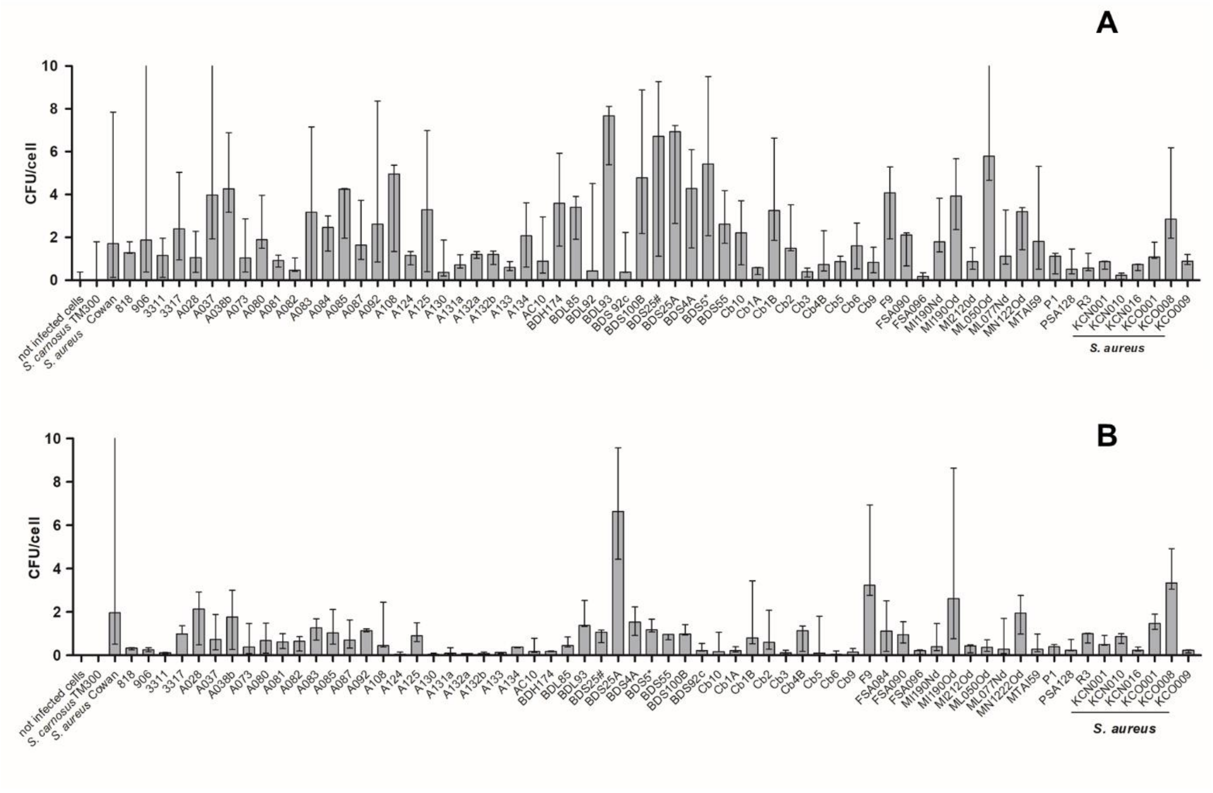

Figure S2: Variance of *Staphylococcus schweitzeri* to invade host cells. Human epithelial cells (A549, A) and monkey kidney cells (Vero, B) were infected with 58 different *S. schweitzeri* isolates and showed a high variance of intracellular bacterial cells. *S. aureus* isolates represent the three most common MLST sequence types (ST) in Africa (two isolates per ST, from colonization and infection). Bars represent the median (range) of three independent experiments.

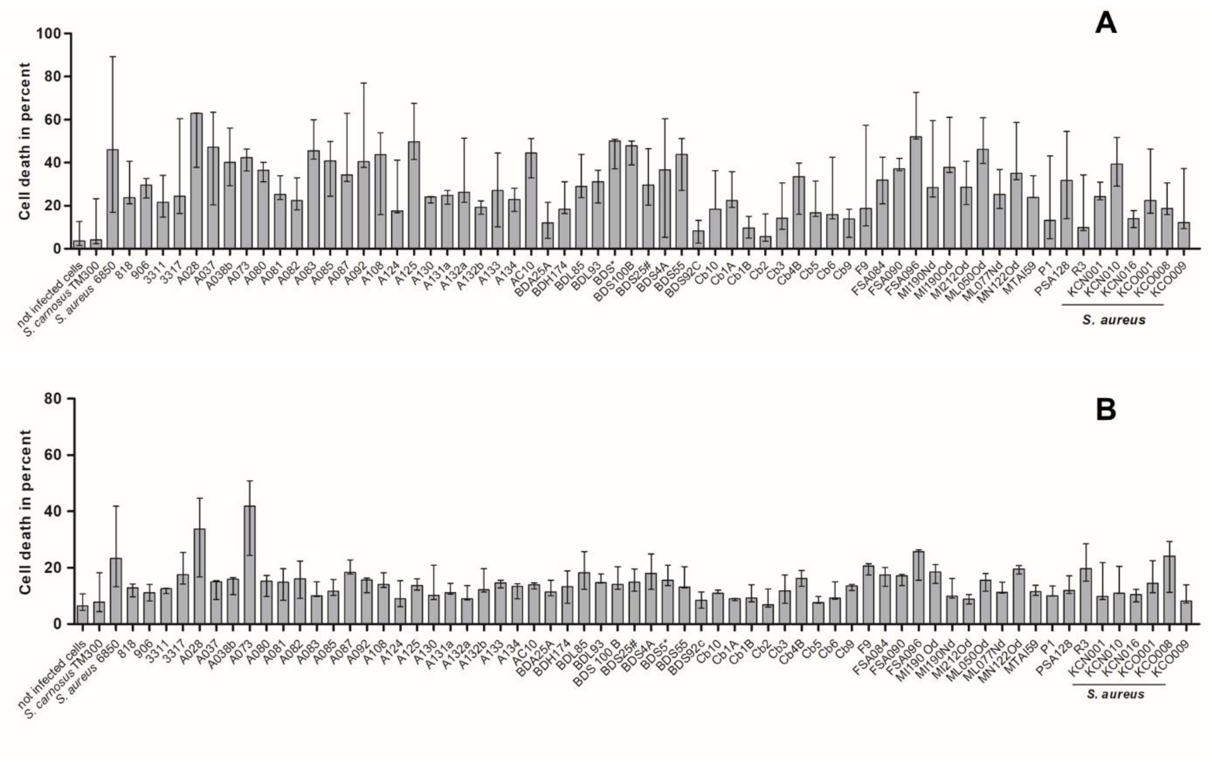

Figure S3: Variance of *Staphylococcus schweitzeri* to induce intracellular cytotoxicity. Human epithelial cells (A549, A) and monkey kidney cells (Vero, B) were infected with 58 different *S. schweitzeri* isolates and showed a high variance of intracellular cytotoxicity. *S. aureus* isolates represent the three most common MLST sequence types (ST) in Africa (two isolates per ST, from colonization and infection). Bars represent the median (range) proportion of dead host cells of three independent experiments.

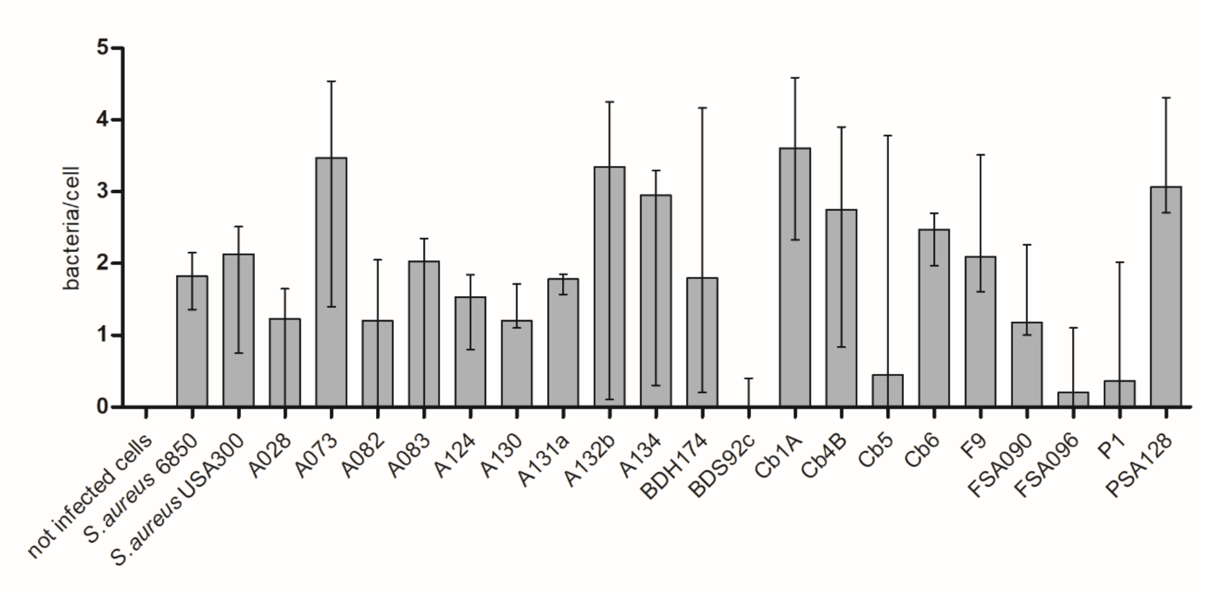

Figure S4: Phagolysosomal escape of *Staphylococcus schweitzeri*. Human epithelial cells (A549) expressing YFP-CWT were infected with twenty randomly selected *S. schweitzeri* isolates. Nearly all tested *S. schweitzeri* (18/20) showed the ability to escape from phagolysosomes with a marked difference between the isolates. Bars represent the median (range) number of escaped bacteria/host cell and range.

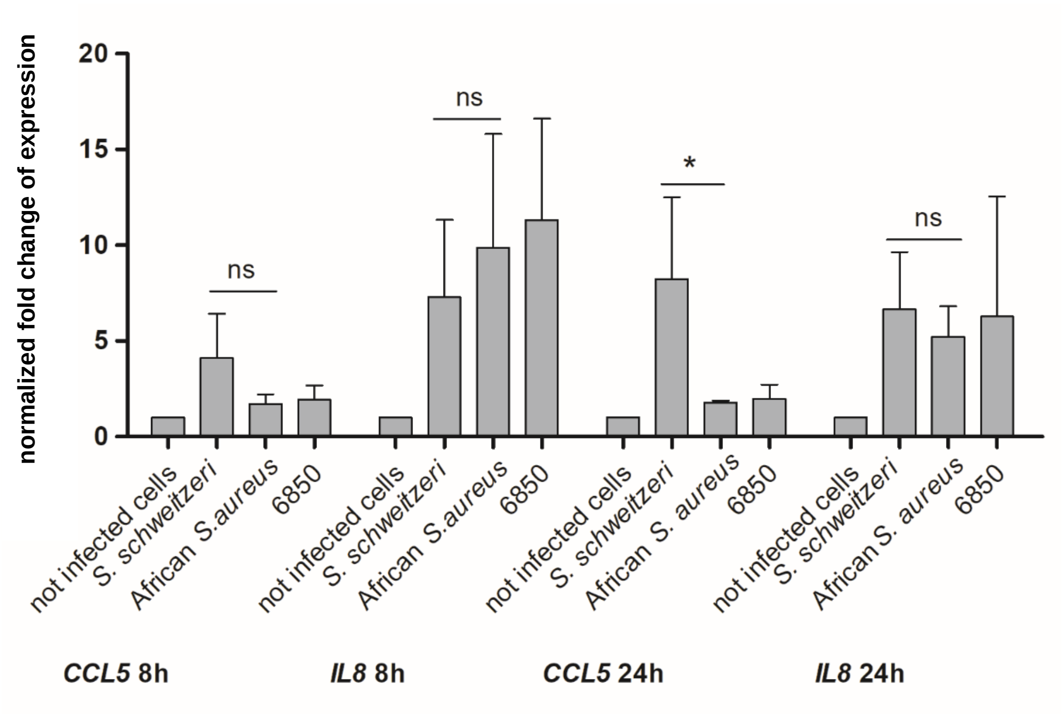

Figure S5: Expression levels of *CCL5* and *IL8*. A549 cells were infected with twenty randomly selected *S. schweitzeri* and the expression of *CCL5* and *IL8* were measured by qRT-PCR. Infection of A549 with *S. aureus* 6850 served as a control. Bars represent the mean (±SD) of normalized fold change of gene expression. The mean of the normalized fold change of *S. schweitzeri* and African *S. aureus* were compared by t-test, as the normalized fold expression was normally distributed. * $p \leq 0.05$, ns = not significant

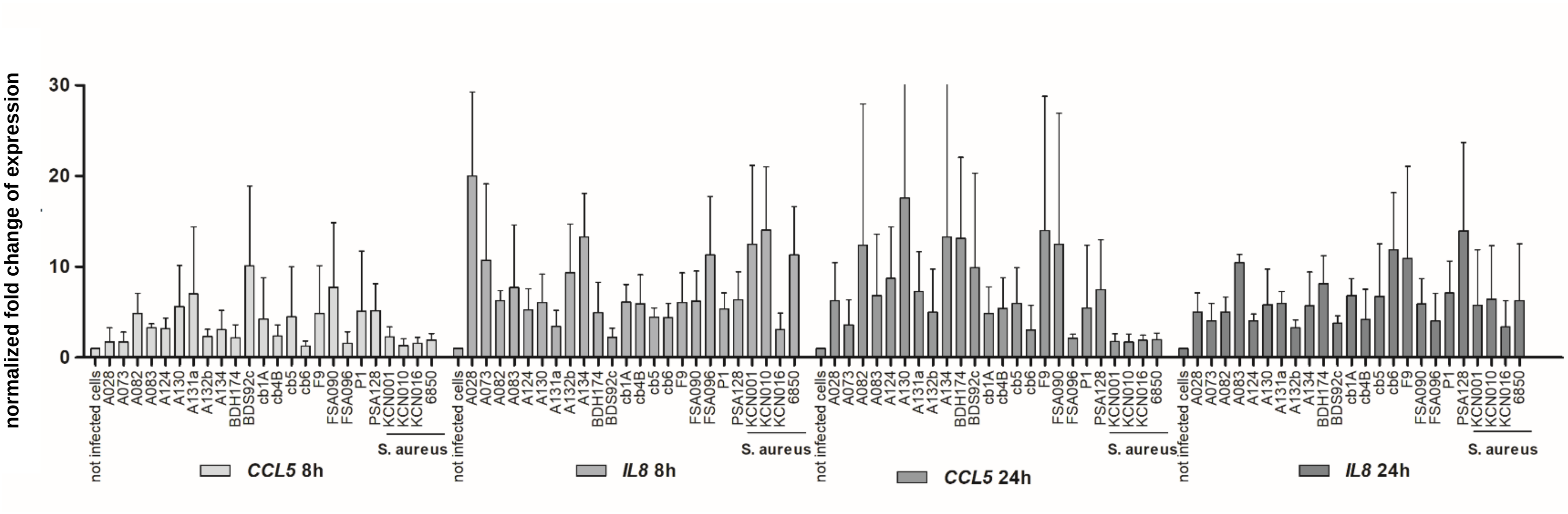

Figure S6: Variance of *Staphylococcus schweitzeri* to induce *CCL5* and *IL8* expression in A549 cells after 8 and 24h. A549 cells were infected with twenty randomly selected *S. schweitzeri* and the expression of *CCL5* and *IL8* were measured by qRT-PCR.

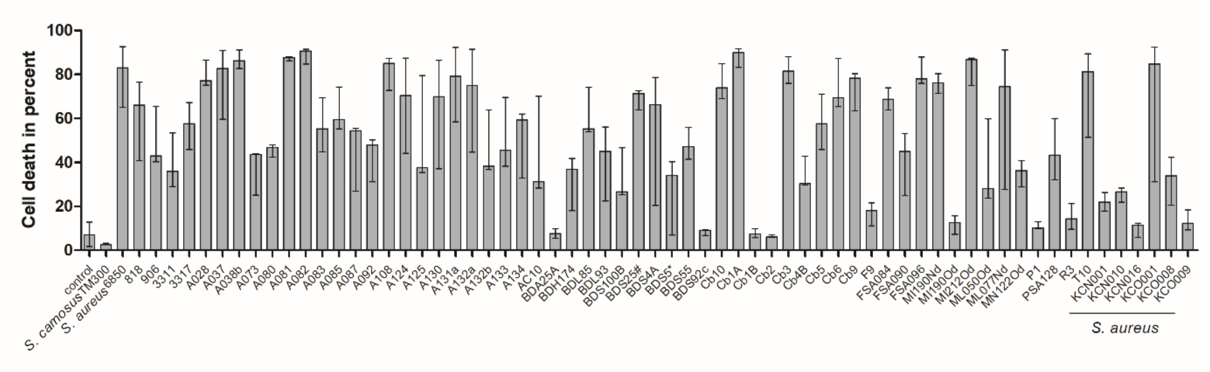

Figure S7: Variance of *Staphylococcus schweitzeri* to induce extracellular cytotoxicity. Human epithelial cells (A549) were incubated with supernatants of 58 different *S. schweitzeri* isolates for 24 hours and the proportion of PI-stained cells was measured. *S. aureus* isolates represent the three most common MLST sequence types (ST) in Africa (two isolates per ST, from colonization and infection). Bars represent the median (range) of three independent experiments.

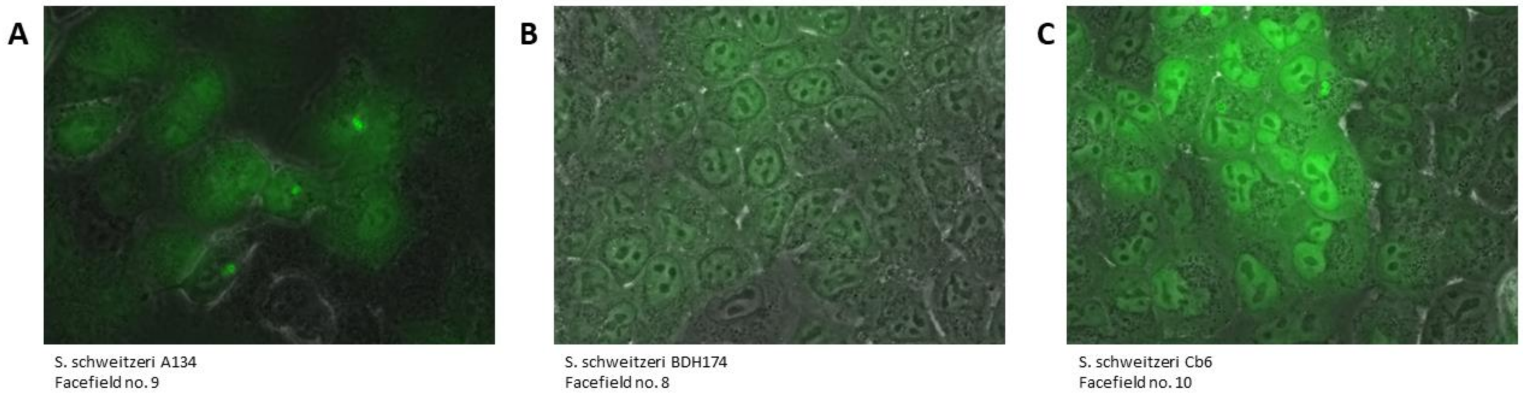

Figure S8: Phagolysosomal escape was quantified by infecting A549 cells, which express the escape marker YFP-CWT in the cytoplasm. YFP-CWT is recruited to the peptidoglycan of the bacterial cell wall upon rupture of the phagolysosomal membrane barrier. Ten fields of view were analysed for each isolate.

References

1 Olatimehin, A. *et al.* *Staphylococcus aureus* Complex in the Straw-Colored Fruit Bat (*Eidolon helvum*) in Nigeria. *Front Microbiol* **9**, 162, doi:10.3389/fmicb.2018.00162 (2018).

2 Schaumburg, F. *et al.* *Staphylococcus aureus* complex from animals and humans in three remote African regions. *Clin Microbiol Infect* **21**, 345 e341-348, doi:10.1016/j.cmi.2014.12.001 (2015).

3 Tong, S. Y. *et al.* Novel staphylococcal species that form part of a *Staphylococcus aureus*-related complex: the non-pigmented *Staphylococcus argenteus* sp. nov. and the non-human primate-associated *Staphylococcus schweitzeri* sp. nov. *Int J Syst Evol Microbiol* **65**, 15-22, doi:10.1099/ijs.0.062752-0 (2015).

4 Akobi, B., Aboderin, O., Sasaki, T. & Shittu, A. Characterization of *Staphylococcus aureus* isolates from faecal samples of the Straw-Coloured Fruit Bat (*Eidolon helvum*) in Obafemi Awolowo University (OAU), Nigeria. *BMC Microbiol* **12**, 1-8, doi:10.1186/1471-2180-12-279 (2012).

5 Ateba Ngoa, U. *et al.* Epidemiology and population structure of *Staphylococcus aureus* in various population groups from a rural and semi urban area in Gabon, Central Africa. *Acta Trop* **124**, 42-47, doi:10.1016/j.actatropica.2012.06.005 (2012).

6 Held, J. *et al.* Bats are rare reservoirs of *Staphylococcus aureus* complex in Gabon. *Infect Genet Evol* **47**, 118-120, doi:10.1016/j.meegid.2016.11.022 (2017).

7 Okuda, K. V. *et al.* Molecular epidemiology of *Staphylococcus aureus* from Lambarene, Gabon. *Eur J Clin Microbiol Infect Dis* **35**, 1963-1973, doi:10.1007/s10096-016-2748-z (2016).

8 Strobel, M. *et al.* Post invasion events after infection with *Staphylococcus aureus* are strongly dependent on both the host cell type and the infecting *S. aureus* strain. *Clin Microbiol Infect* **22**, 799-809, doi:http://dx.doi.org/10.1016/j.cmi.2016.06.020 (2016).

9 Voss, A. *et al.* Expression of S100A8/A9 in HaCaT keratinocytes alters the rate of cell proliferation and differentiation. *FEBS Letters* **585**, 440-446, doi:10.1016/j.febslet.2010.12.037 (2011).
